# Supplementary material for: Highly dynamic mechanical transitions in embryonic cell populations during Drosophila gastrulation
Source: Nat Commun. 2025 Jul 14;16:6473. doi: 10.1038/s41467-025-61702-4 (PMC12259986; doi:10.1038/s41467-025-61702-4)
Supplement: Supplementary file 1 — Supplementary Information [file 41467_2025_61702_MOESM1_ESM.pdf]

Supplementary Information:

Highly dynamic mechanical transitions in embryonic cell populations during *Drosophila* gastrulation

Juan Manuel Gomez[1*], Carlo Bevilacqua[1], Abhisha Thayambath[2,3], Jean-Karim Heriche[1], Maria Leptin[4,5,6], Julio M Belmonte[2,3] and Robert Prevedel[1,4,7,8*].

[1] Cell Biology and Biophysics Unit, European Molecular Biology Laboratory (EMBL), Heidelberg, Germany.
[2] Quantitative and Computational Developmental Biology Cluster, North Carolina State University, Raleigh, North Carolina, United States of America.
[3] Department of Physics, North Carolina State University, Raleigh, North Carolina, United States of America.
[4] Developmental Biology Unit, European Molecular Biology Laboratory (EMBL), Heidelberg, Germany.
[5] Director's research, European Molecular Biology Laboratory (EMBL), Heidelberg, Germany.
[6] Institute of Genetics, University of Cologne, Cologne, Germany.
[7] Epigenetics and Neurobiology Unit, European Molecular Biology Laboratory (EMBL), Rome, Italy.
[8] German Center for Lung Research (DZL), Heidelberg, Germany.

* Co-corresponding authors: juan.elliff@embl.de; prevedel@embl.de

Supplementary Figure 1

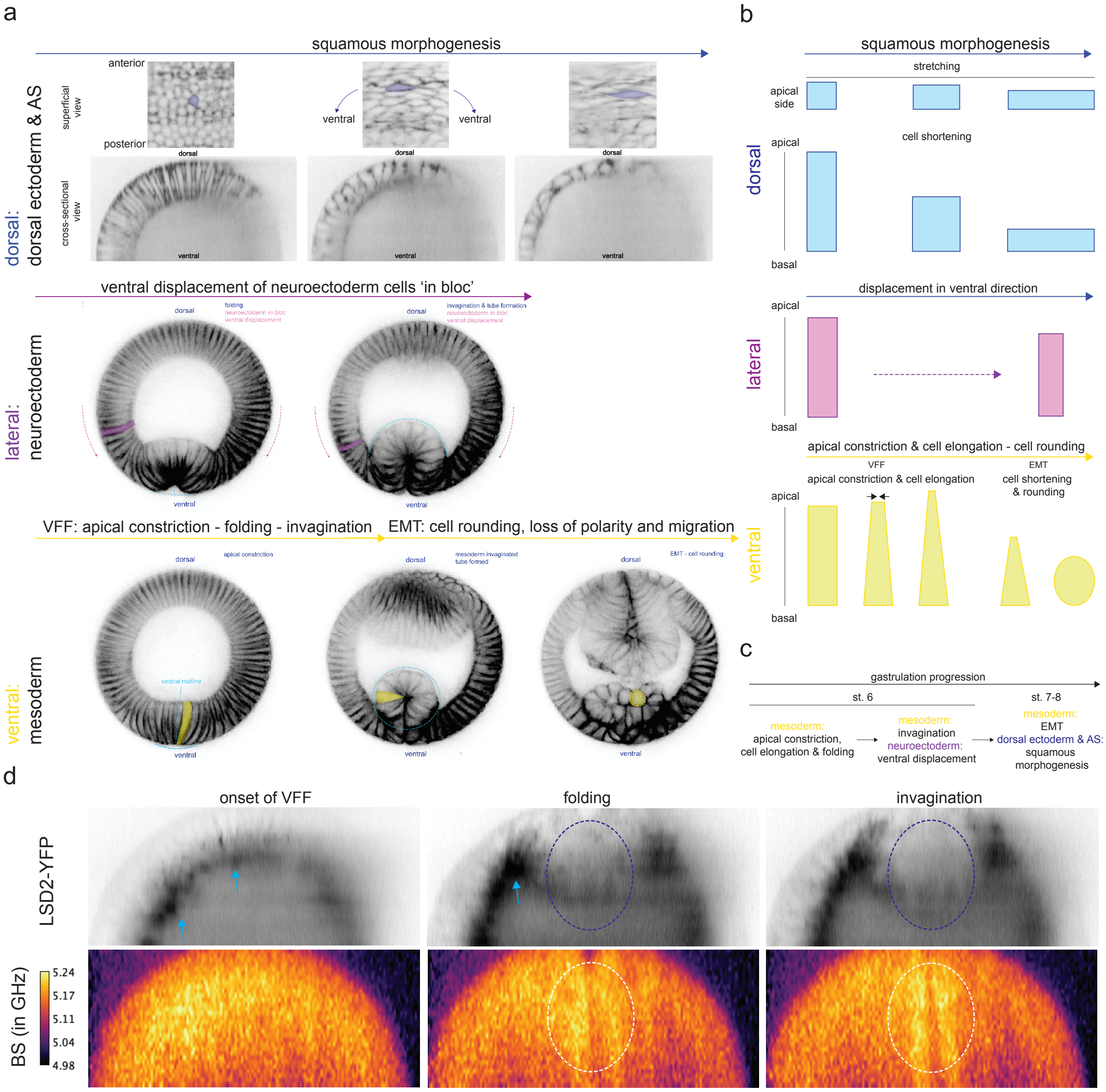

Cell shape behaviours in cells along the dorso-ventral axis during *Drosophila* gastrulation.

a. Description of cell shape behaviours within each DV cell population. Top: dorsal side of the embryo, showing squamous morphogenesis in dorsal ectoderm and amnioserosa (AS) cells. The process of squamous morphogenesis leads to the stretching of cells (along the embryonic DV axis, anterior is top, blue shaded cell) and the shortening of cells (along the cellular apical-basal axis). Lateral and Ventral: 2-photon cross-sectional imaging of a *Drosophila* embryo showing cell shape behaviours within the Neuroectoderm (lateral, magenta shaded cell) and Mesoderm (ventral, yellow shaded cells). Neuroectoderm cells display little changes in their cellular geometry; Mesoderm cells undergo apical constriction and cell lengthening (during VFF, left panel), which is followed by cell shortening and rounding during EMT (centre and right panels respectively). In all cross-sectional panels, dorsal is top, ventral is bottom.

b. Illustration that schematises the changes in cellular geometry in the dorsal, lateral and ventral side of the embryo.

c. Coordination of morphogenetic events within DV cell populations (yellow: mesoderm; magenta: neuroectoderm; blue: dorsal ectoderm and AS) during gastrulation (starting in stage 6, and continued during stage 7 and 8), based on work by Rauzi et al. [1].

d. Colocalisation between a protein trap for *lsd-2*, lsd2-YFP (top panel), and the transient increase in Brillouin shift (BS in GHz, bottom panel) that is measured within the mesoderm during VFF. Lsd2 associates with lipid droplets and vesicles. Area of the mesoderm encircled with a blue and white-dashed line shows increase in the Brillouin shift during folding and invagination. Light-blue arrows indicate Lsd2-YFP signal on the central or peripheral mesoderm. Images are average projections of two consecutive YZ re-slices of the original volume. Note that Lsd2-YFP puncta do not colocalise with the high Brillouin shift in central mesoderm.

Supplementary Figure 2

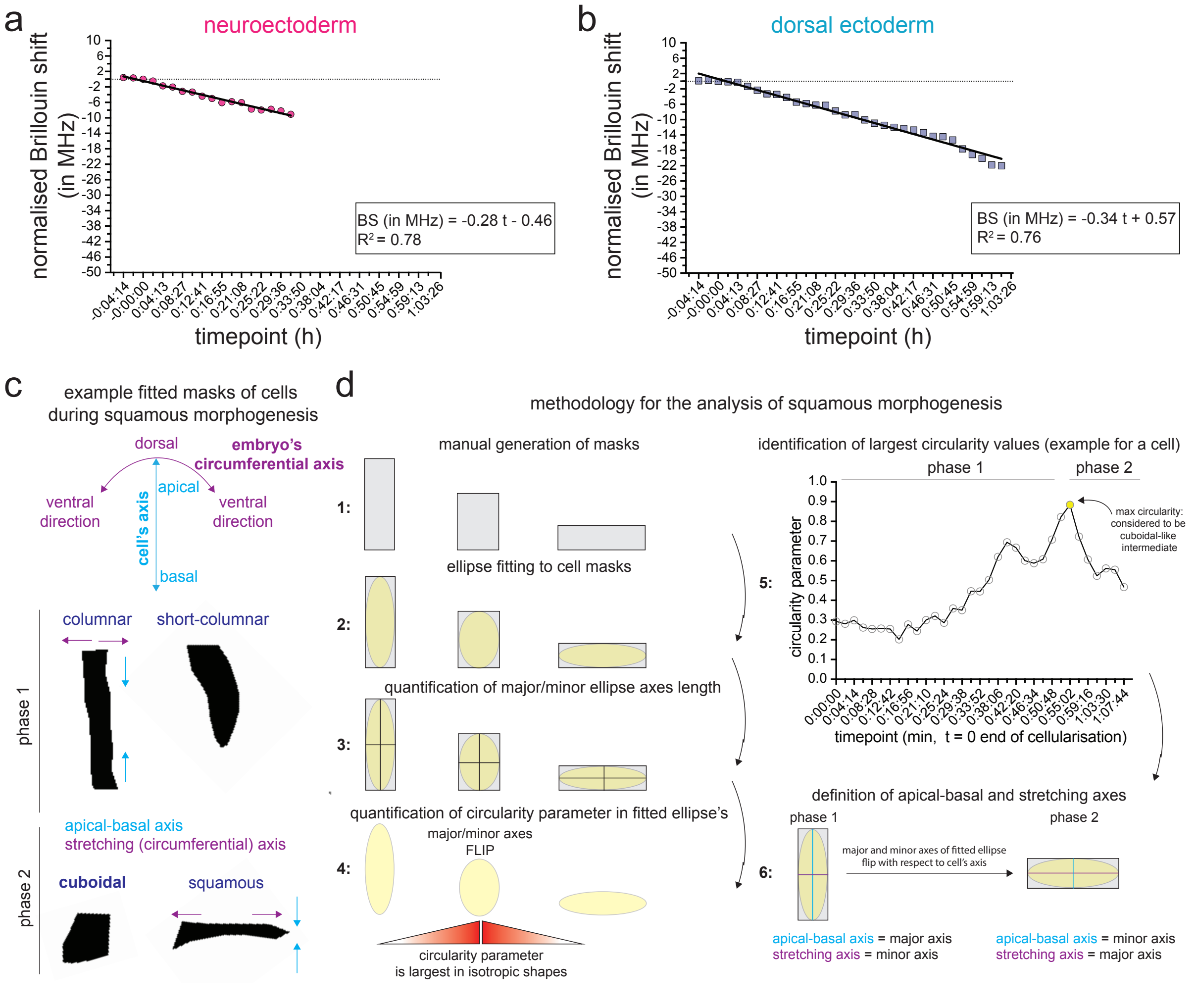

Complementary and supporting results to the analyses of squamous morphogenesis.

a. Mean (N = 5) BS dynamics in the neuroectoderm (in magenta; lateral cells; Fig. 1d,e) during gastrulation fitted with a linear regression: BS (in MHz) = -0.28 t - 0.46; R^2 = 0.78.

b. Mean (N = 5) BS dynamics in the dorsal ectoderm (in blue; dorsal cells; Fig. 1f,g) during gastrulation fitted with a linear regression: BS (in MHz) = -0.34 + 0.57; R^2 = 0.76.

c. Description of progressive changes in cell geometry during the squamous morphogenesis of dorsal cells (dorsal ectoderm and AS) at gastrulation stage. Shapes are examples of manually-generated cell masks of a dorsal cell of an embryo at gastrulation stage. Right panel shows the axes in which changes in cell geometry occur: cell apical-basal axis (cyan) and along the perimeter of the embryo in the dorso-ventral direction (magenta). During squamous morphogenesis cells shorten along the apical-basal axis and stretch in the ventral

direction. We defined two phases during squamous morphogenesis, *phase 1* prior to the formation of the 'cuboidal' intermediate, and *phase 2*, subsequently.

d. Illustration that describes key steps (1-6) of the methodology used to analyse the cellular shapes during squamous morphogenesis. Cells that could be followed in a single reslice of the acquired volume from the end of cellularisation until cells became squamous were manually segmented (step 1). The measurements of the major and minor axes length (step 2-3) were used to quantify the apical-basal (cyan) and stretching axes (magenta, perpendicular to apical-basal axis). To detect the 'cuboidal' intermediate we used the circularity parameter, which is larger when cells are increasingly isotropic (steps 4-5, yellow dot in circularity parameter timelapse). The cell mask with the maximum circularity value was considered to represent the 'cuboidal' intermediate, and the boundary between phase 1 and 2. During phase 1, the major axis length was used to measure the apical-basal axis and the minor axis was used to measure the stretching axis. During phase 2, the major axis length was used to measure the stretching axis, and the minor axis was used to measure the apical-basal axis.

Source data are provided as a Source Data file.

Supplementary Figure 3

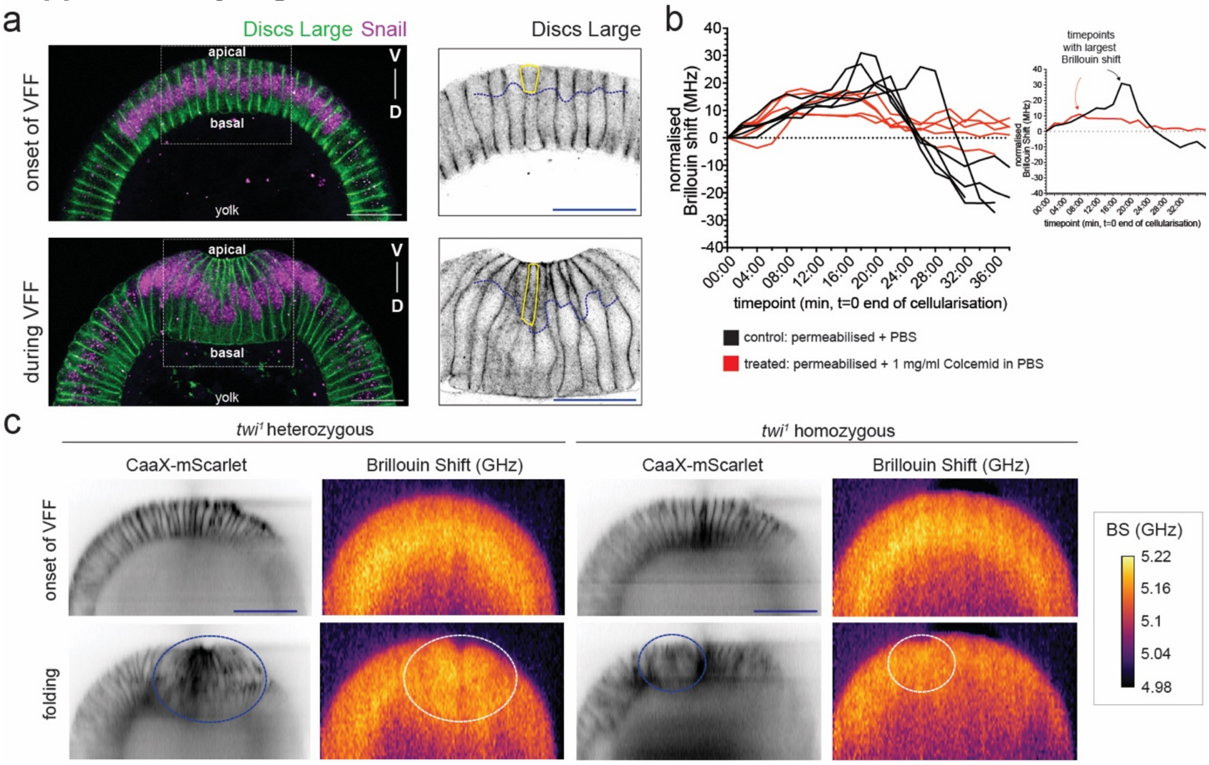

Complementary and supporting results for the analyses of the mechanical role of Microtubules during VFF

a. Visualisation of cell shape (Discs Large: green) and nuclei (Snail: magenta) in the mesoderm, before (top panel) and during ventral furrow formation (VFF) from physical cross-sections of fixed embryos. Dorsal (D) is bottom, ventral (V) is top. Insets (corresponding to white-dashed squares in full-images) show cell shapes in central mesodermal cells (Discs Large: grayscale). Blue dashed line indicates the apical boundary of nuclei (Snail-positive signal). Yellow line encases the sub-apical compartment before (top inset) and during VFF (bottom inset). Images correspond to representative images in one experiment, among a total of three independent experiments (N=3). Scale bars are 25 μm.

b. Quantification of the BS within 6 central mesoderm cells (3 cells on each side of the ventral midline, see Supplementary Fig. 1a, bottom panel and Supplementary Movie 1) during VFF in

permeabilised embryos treated with Colcemid 1 mg/ml in PBS or left untreated (control, PBS). Each line is a single embryo and an independent experiment (N=5 for each condition). Control embryos are shown with black lines, Colcemid-treated embryos are shown with red lines. Quantifications were performed in median projections of three consecutive YZ reslices of the acquired raw volume. BS was normalised to the onset of VFF (t = 0'). Right inset shows a control and a Colcemid-treated embryo, and black/grey arrows indicate the maximum BS value that was used to perform the quantification of the effect of Colcemid treatment on the transient increase in BS during VFF (Fig. 4f,g).
c. Brillouin shift maps in *twist* (*twi*^{1}, right panel) mutant embryos during VFF. *twist* mutants engage a smaller number of cells that in control embryos (*twi*^{1}/+, left panel), form a smaller fold and fail to invaginate the mesoderm; note the transient high Brillouin shift is strongly reduced in *twi*^{1}. Blue and white dashed-line circles indicate the area of the blastoderm engaged in the folding event. Representative images from the live imaging of an embryo. N = 3 independent experiments. Scale bar is 50 µm. Source data are provided as a Source Data file.

Supplementary Figure 4

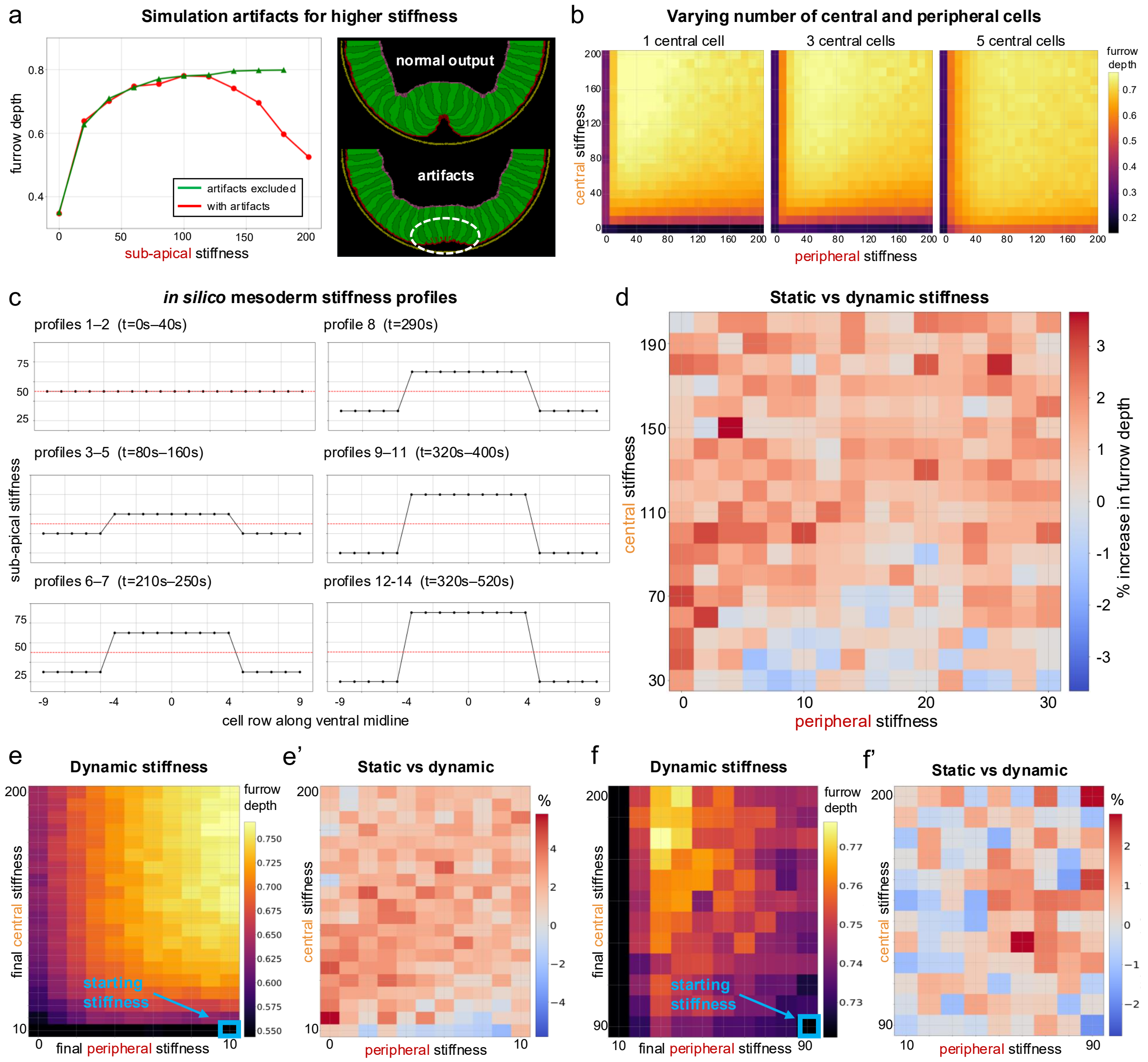

Complementary and supporting results for the physical model of VFF
a. Furrow depth as a function of sub-apical longitudinal stiffness (sub-basal stiffness set to 40 a.u.). For low values of longitudinal stiffness the model behaves normally and furrow

depth correlates with higher stiffness. When stiffness is increased beyond 100 a.u. the model starts presenting artefacts and results can no longer be trusted.

b. Effect of different definitions for the number of central vs peripheral cells in the results about the role of central vs peripheral cell stiffness in furrow formation. Results remain the same when a similar definition for the number of central cells as in Fig. 5e is used.

c. Set of 5 sub-apical longitudinal stiffness profiles along the ventral midline used in our simulations (Fig. 5f and Fig S4). Cells start with uniform stiffness throughout the mesoderm, with an initial softening of the peripheral cells (profiles 3 to 4), followed by a stiffening of the central cells (profiles 5 to 9). The maximum and minimum values of central and peripheral cell stiffness is different for each simulation scenario (Fig. 5f).

d. Quantitative comparison between static (Fig. 5e) and dynamic stiffness (Fig. 5f) on furrow formation. Red colours represent an increased furrow depth for dynamic stiffness compared to the static case.

e. Furrow depth as a function of increasing sub-apical central cell longitudinal stiffness and decreasing sub-apical peripheral cell stiffness for a starting stiffness value of 10.

e'. Quantitative comparison between static (Fig. 5e) and dynamic stiffness (Fig. 5f) on furrow formation for a starting stiffness value of 90.

f. Furrow depth as a function of increasing sub-apical central cell longitudinal stiffness and decreasing sub-apical peripheral cell stiffness for a starting stiffness value of 10.

f'. Quantitative comparison between static (Fig. 5e) and dynamic stiffness (Fig. 5f) on furrow formation for a starting stiffness value of 90.

Source data are provided as a Source Data file.

Supplementary Figure 5

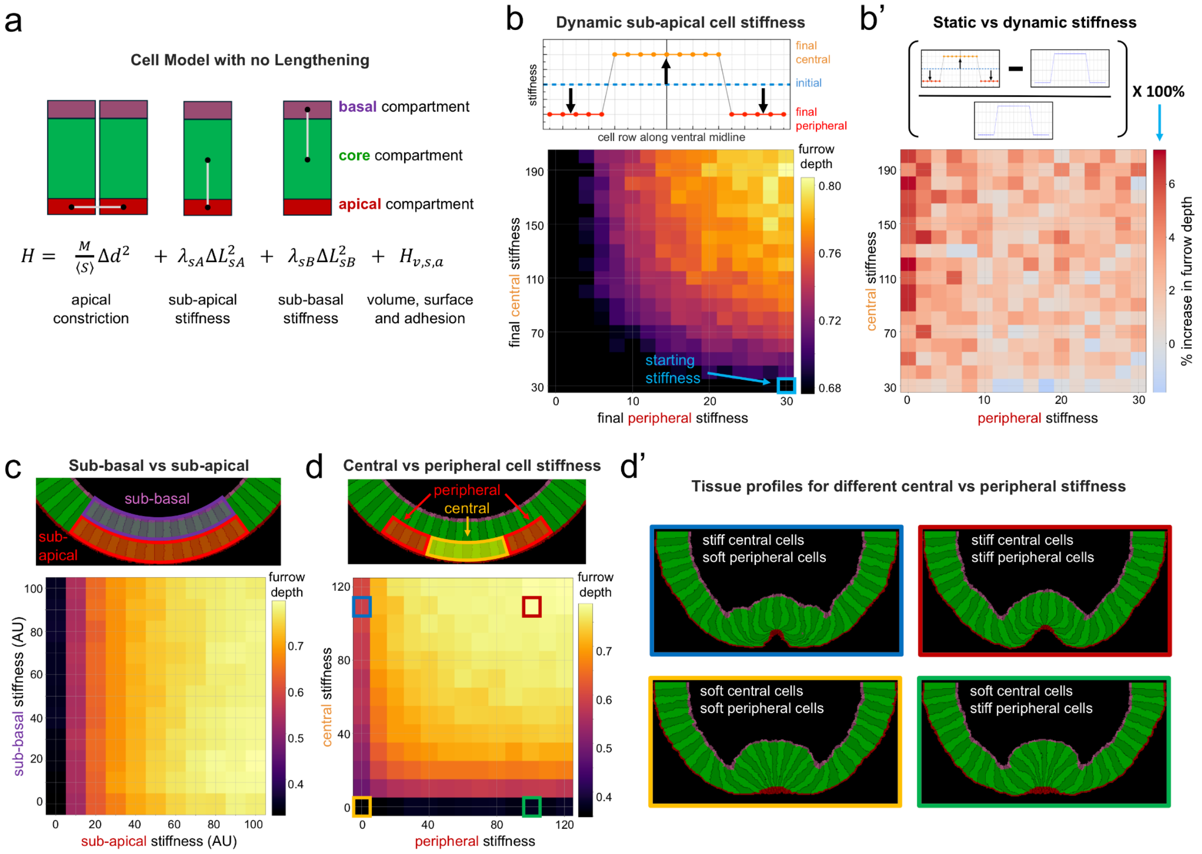

Results for the physical model of VFF without cell lengthening

a. Each cell was modelled with 3 compartments: apical, core and basal. Hookean links connecting the centre of mass of neighbouring compartments are used to model apical constriction between adjacent mesodermal cells, and sub-apical and sub-basal longitudinal stiffness of mesodermal cells. Those constraints are part of an effective energy equation (*H*)

that guides the model evolution and also include an adhesion, surface and volume terms as indicated.
b. Furrow depth as a function of increasing sub-apical central cell longitudinal stiffness and decreasing sub-apical peripheral cell stiffness. Grid coordinate indicates final values of central and peripheral cell stiffness. Colour bar cropped at the furrow depth value of simulations where stiffness of both cell populations remains constant at 30. Sub-basal stiffness was kept constant and the same for both cell populations. Each grid point value shows the average of 30 simulations.
b'. Quantitative comparison between static (**d**) and dynamic stiffness (**b**) on furrow formation. Red colours represent an increased furrow depth for dynamic stiffness compared to the static case.
c. Furrow depth as a function of sub-apical and sub-basal longitudinal stiffness of mesodermal cells. Sub-basal stiffness variation has little effect on furrow formation, while higher values of sub-apical stiffness lead to deeper furrows. Each grid point value shows the average of 30 simulations.
d. Furrow depth as a function of sub-apical longitudinal stiffness of central and peripheral cells. When either cell population is too soft, furrows fail to form (c.f. examples in panel **d'**). Higher values of central cell stiffness promote furrow formation even for relatively low values of peripheral cell stiffness. Sub-basal stiffness was the same for both cell populations. Each grid point value shows the average of 30 simulations.
d'. Representative simulation snapshots of 4 cases from panel **d** (indicated by corresponding outline colour).
Source data are provided as a Source Data file.

Supplementary Note 1

Our physical model of *Drosophila* VFF was developed within the Cellular Potts (aka Glazier-Graner-Hogeweg) modelling framework [2] using the CompuCell3D (CC3D) simulation software [3]. In this modelling framework, biological cells and their subcellular compartments are spatially represented as a collection of lattice sites in a regular (cartesian) grid with the same ID. Our model consists of a 2D cross-section of the ventral half of the *Drosophila* embryo, consisting of 47 cells, including 19 mesodermal cells and 28 neuroectodermal cells (Fig. 5a). Each cell is composed of at least three compartments: **apical, core** and **basal**, reflecting the apical–basal polarity of blastoderm with a vitelline membrane surrounding the cells. To simulate the observed heightening of the cells during VFF, we introduce an additional domain between the basal and core compartments. This domain called **core2** compartment grows over time at a rate *delta* for mesodermal cells and *delta/4* for neuroectodermal cells and has all the physical properties (adhesion preferences, volume constraints etc.) of the core compartment. Addition of this growing/heightening mechanism greatly improves the realism and final shapes of the tissue, while maintaining most of the qualitative results from a model version without this mechanism (see Supplementary Fig 5).

An effective energy equation (H_{Total}) defines cell/domain properties and behaviours such as size, aspect-ratios, adhesion preferences and interactions with other cells (Eq. 1). Each of these properties is governed by an individual term in the overall effective energy equation: a volume constraint (H_V) helps maintain the size and compressibility of cell compartments; a surface constraint (H_S) helps maintain the perimeter of apical compartments; a contact energy (H_C) specifies the relative strength of contact/adhesion between compartments from the same and/or different cells; and a spring-like intercellular force term (H_F) linking compartments' centre-of-mass can be used to model apical constriction between mesodermal cells and sub-apical and sub-basal longitudinal stiffness of cells by linking apical-core and core-basal cell compartments, respectively.

(Eq. 1) $$H_{Total} = H_V + H_S + H_C + H_F$$

The system evolves by a series of random lattice-site copy attempts where a lattice site "i" is randomly selected, and a neighbouring lattice site "j" is randomly selected within the 4th neighbour order of "i". If the two lattice sites belong to different cells or cell compartments, we evaluate the difference in energy (ΔH) if the ID of site "i" is copied over site "j". If the effective energy is reduced ($\Delta H<0$) the copy is accepted; if it is increased then the copy attempt is accepted with a probability equal to:

(Eq. 2) $\exp(-\Delta H/T)$

where T is a parameter used to specify the level of fluctuations in the system. A monte-carlo step (MCS) is defined as N lattice-site copy attempts, where N is the number of lattice sites in the grid. In our model 1000 MCS corresponds to 20 seconds.

The volume constraint is used to penalise small deviations of cell compartments volume (v) – which in a 2D simulation is defined as the number of lattice sites occupied by the cell compartment – from their target volume (v_t):

(Eq. 3) $H_V = \lambda_V(v-v_t)^2$

where λ_V is parameter proportional to the bulk modulus of the cell compartment.

The surface constraint is used to penalise small deviations of cell compartments surface (s) – which in a 2D simulation is defined as the total perimeter that the occupied lattice sites of the cell compartment has – from their target surface (s_t):

(Eq. 3) $H_S = \lambda_S(s-s_t)^2$

where λ_S is a parameter setting the strength of the constraints. This constraint is only used for apical compartments to prevent their fragmentation during apical constriction.

The contact energy (H_C) is modelled as:

(Eq. 4) $H_c = \sum_i \sum_{j\neq i} J(\sigma_i, \sigma_j)$

where the second sum is over the 4th neighbourhood of site "i"; σ_i and σ_j are the type of cell compartments occupying sites "i" and "j", respectively; and J is a matrix containing the contact energy between compartments belonging to the same cell and different cells.

The spring-like intercellular force constraint (H_F) has the form of:

(Eq. 5) $H_F = \lambda_F(d-d_t)^2$

where d is the distance between two cell compartments centre-of-mass (defined by the spatial distribution of the lattice sites it currently occupies in the grid); d_t is the target distance between them; and λ_F is the strength of the constraint. A different H_F, with its own parameters λ_F and d_t is defined for each pair of cell compartments that are linked by such a force.

Assuming a linear relation between the longitudinal stiffness and the longitudinal strain, we use (Eq. 5) to model the longitudinal stress/strain relation of the mesodermal cells along the apical-basal axis by linking their internal compartments:

(Eq. 6a) $H_{sA} = \lambda_{sA}(d_{a\text{-}c}-d_{a\text{-}c,t})^2$

(Eq. 6b) $H_{sB} = \lambda_{sB}(d_{c2c}-d_{c2\text{-}c,t})^2$

where the $d_{a\text{-}c}$ and $d_{c2\text{-}c}$ is the distance between the centre of mass of the apical and core compartments, and the core2 and core compartments, respectively; $d_{a\text{-}c,t}$ is the initial distance between apical-core compartments at the start of the simulation whereas $d_{c2\text{-}c,t}$ is the distance between core-core2 compartments which changes over time as the cell heightens; λ_{sA} and λ_{sB}

are parameters proportional to the longitudinal modulus (*M*) of the sub-apical and sub-basal regions of the modelled cells (which we will now on simply refer as the "stiffness" of the compartments).

The contractile force generated as a result of medial-apical actomyosin accumulation is implemented in the model using spring-like forces that link the 19 central mesodermal cells at apical sides (H_A). This constraint is added to the effective energy as

(Eq. 7) $$H_A = (M/< S >)(d-d_t)^2$$

where the *d* is the distance between the centre of mass of the two apical compartments; d_t is the target distance between these compartments (set to 1 lattice site); and the ratio M/<S> is the strength of the contractile force, where M is the time-varying myosin levels and <S> is the average apical surface area with the medium of the two linked cells. We utilised measured myosin levels dynamics within the mesoderm [4] to construct the time-varying profiles of the myosin levels in our simulations and to set the time stamp of the simulations.

To measure the success of VFF we used furrow depth (Fig. 5a) as a metric to quantify the extent of fold formation and, therefore, as a proxy for the chance of successful invagination. This is a common metric used in in many experiments and simulations of gastrulation [1] [5] [6] [7] [8] that takes into account the observed correlation between smaller or shallow mesoderm folds with failed invagination [9] [10] [11]. Furrow depth in our simulations is measured as the normalised distance between the apical-most coordinate of the central mesodermal cell and the lowest point of the mesoderm from the initial condition in units of initial cell height in the apical-basal axis (Fig. 5a). All measurements of furrow depth reported in the parameter's maps of Figs. 5d-e are averages of 10 to 30 simulations.

For most of our analysis longitudinal cell stiffness was varied over a range from 0 to about 160 (values in arbitrary units), with higher values always leading to deeper furrows. In our model, increasing stiffness beyond those values lead to simulation artefacts such as shown in Supplementary Fig. 4a and we chose to exclude those cases from our analysis. Because of that we were unable to comment on how higher levels of stiffness may also prevent furrow formation by not allowing for any cell deformation.

For the analysis of the impact of stiffness gradient along the ventral mesoderm we split our modelled mesoderm between 9 central cells and 10 peripheral cells (5 on each side). This was done to simplify our analysis and the choice of 9 central cells was done to reflect the measured point of inflection in stiffness value measured in the experiments. Choosing a slight lower number of central cells (5) had very little quantitative effect in our results (Supplementary Fig. 4b last panel). It is only when the number of central cells is defined to be lower than 3 that we see a drastic qualitative difference in our results (Supplementary Fig. 4b first panel).

For the simulations with varying cell stiffness over time, we assumed constant sub-basal stiffness (λ_{sB}=50) for all cells and the same initial value of sub-apical stiffness for central and peripheral cells ($\lambda_{sA,initial}$). Over time, the central cell increase their stiffness to a final value ($\lambda_{sA,central\text{-}final}$), while the peripheral cells decrease their stiffness to a final value ($\lambda_{sA,peripheral\text{-}final}$), according to the evolution shown in Supplementary Fig. 4c. Choosing a higher(lower) starting value ($\lambda_{sA,initial}$) for the cell stiffness leads to less(more) prominent results regarding the gain of furrow depth for dynamic changes in stiffness and also for the static vs dynamic comparison (see Supplementary Fig. 4e,f).

For the quantitative comparison between static and dynamic stiffness (Supplementary Fig 4d) we calculated the % increase in furrow depth from the static case (F_S, when the cells already start with the central and peripheral stiffness indicated by the coordinate values) and the dynamic case (F_D, when all cells start with a uniform stiffness of 30, and modify their central and peripheral cell stiffness over time until the value indicated by the grid coordinates). The formula used was $100\%x(F_D-F_S)/F_S$.

Table S1: List of parameters used in the model

Parameter	Name	Value
T	**CPM fluctuation amplitude**	25
n_copy	**Neighbor range for lattice site copy attempts**	2
n_contact	**Neighbor range for contact energy calculations**	4
t	**Total time**	26000 MCS
λ_s	**<u>Strengh of Surface Constraint</u>**	
	Strength of Apical(mesoderm) surface constraint	1
**For the model with cell lengthening**		
λ_v	**<u>Strength of Volume Constraint</u>**	
	Apical(mesedorm)	2
	Apical(neuroectoderm)	100
	Core(mesoderm)	10
	Core(neuroectoderm)	100
	Basal(mesoderm)	15
	Basal(neuroectoderm)	100
	Core2(mesoderm)	10
	Core2(neuroectoderm)	10
	Vitelline membrane(VM)	1000
	**<u>Apical Constriction</u>**	
d_t	Target link length for apical links between apical domains in neighbouring cells	1 pixel
M_min	Minimum strength of contractile force(myosin level)	1
M_multiplier	Peak value multiplier for contractile force(myosin level)	10
t_relax	Relaxation time after each myosin profile	2000 MCS
	**<u>Cell Stiffness Parameters</u>**	

λ_sB	Strength of Core2-Core internal link(sub-basal stiffness)	[0,10,20,...190,200]
λ_sA	Strength of Apical-Core internal link(sub-apical stiffness)	[0,10,20,...190,200]
J	**Contact Energies(External)**	
	medium-medium	0
	medium-apical	8
	medium-core	100
	medium-basal	8
	medium-core'	100
	medium-basal'	8
	medium-vm	8
	medium-core2	100
	medium-core2'	100
	apical-apical	10
	apical-core	100
	apical-core'	100
	apical-basal	100
	apical-basal'	100
	apical-vm	50
	apical-core2	100
	apical-core2'	100
	core-core'	10
	core-core	100
	core'-core'	100
	core-basal	100
	core-basal'	100
	core'-basal	100
	core'-basal'	100
	core-vm	100
	core'-vm	100
	core-core2	100
	core'-core2'	100

	core-core2'	10
	core'-core2	10
	basal-basal'	5
	basal-basal	100
	basal'-basal'	100
	basal-vm	100
	basal'-vm	100
	basal'-core2	50
	basal-core2'	50
	basal'-core2'	100
	basal-core2'	100
	vm-vm	0
	vm-core2	100
	vm-core2'	100
	core2-core2'	10
	core2-core2	100
	core2'-core2'	100
	**Contact Energies(Internal)**	
	basal-core	100
	apical-basal	100
	apical-core	2
	basal'-core'	100
	apical-basal'	100
	apical-core'	2
	apical-core2	100
	core-core2	2
	basal-core2	2
	apical-core2'	100
	core'-core2'	2

	basal'-core2'	2
	basal'-vm	100
delta	**Rate of growth for new-basal compartment/growth domain**	
	Mesodermal cells	0.06
	Neuroectodermal cells	0.06/4
**For the model without cell lengthening**		
λ_v	**Strength of Volume Constraint**	
	Apical(mesedorm)	2
	Apical(neuroectoderm)	100
	Core(mesoderm)	10
	Core(neuroectoderm)	100
	Basal(mesoderm)	15
	Basal(neuroectoderm)	100
λ_s	**Strengh of Surface Constraint**	
	Strength of Apical(mesoderm) surface constraint	1
	**Apical Constriction**	
d_t	Target link length for apical links between apical domains in neighbouring cells	1 pixel
M_min	Minimum strength of contractile force(myosin level)	1
M_multiplier	Peak value multiplier for contractile force(myosin level)	15
t_relax	Relaxation time after each myosin profile	2000 MCS
	**Cell Stiffness Parameters**	
λ_sB	Strength of Basal-Core internal link(sub-basal stiffness)	[0,10,20,...190,200]
λ_sA	Strength of Apical-Core internal link(sub-apical stiffness)	[0,10,20,...190,200]
J	**Contact Energies(External)**	
	medium-medium	0

	medium-apical	8
	medium-core	100
	medium-basal	8
	medium-core'	100
	medium-basal'	8
	apical-apical	10
	apical-core	100
	apical-core'	100
	apical-basal	100
	apical-basal'	100
	core-core'	10
	core-core	100
	core'-core'	100
	core-basal	100
	core-basal'	100
	core'-basal	100
	core'-basal'	100
	basal-basal'	5
	basal-basal	100
	basal'-basal'	100
	**Contact Energies(Internal)**	
	basal-core	2
	apical-basal	100
	apical-core	2
	basal'-core'	2
	apical-basal'	100
	apical-core'	2

References

1. Rauzi, M., et al., *Embryo-scale tissue mechanics during Drosophila gastrulation movements.* Nat Commun, 2015. **6**: p. 8677.
2. Graner, F. and J.A. Glazier, *Simulation of biological cell sorting using a two-dimensional extended Potts model.* Phys Rev Lett, 1992. **69**(13): p. 2013-2016.
3. Swat, M.H., et al., *Multi-scale modeling of tissues using CompuCell3D.* Methods Cell Biol, 2012. **110**: p. 325-66.
4. Bhide, S., et al., *Mechanical competition alters the cellular interpretation of an endogenous genetic program.* J Cell Biol, 2021. **220**(11).
5. Conte, V., J.J. Munoz, and M. Miodownik, *A 3D finite element model of ventral furrow invagination in the Drosophila melanogaster embryo.* J Mech Behav Biomed Mater, 2008. **1**(2): p. 188-98.
6. Conte, V., et al., *Robust mechanisms of ventral furrow invagination require the combination of cellular shape changes.* Phys Biol, 2009. **6**(1): p. 016010.
7. Conte, V., et al., *A biomechanical analysis of ventral furrow formation in the Drosophila melanogaster embryo.* PLoS One, 2012. **7**(4): p. e34473.
8. Fierling, J., et al., *Embryo-scale epithelial buckling forms a propagating furrow that initiates gastrulation.* Nat Commun, 2022. **13**(1): p. 3348.
9. Leptin, M. and B. Grunewald, *Cell shape changes during gastrulation in Drosophila.* Development, 1990. **110**(1): p. 73-84.
10. Ko, C.S., V. Tserunyan, and A.C. Martin, *Microtubules promote intercellular contractile force transmission during tissue folding.* J Cell Biol, 2019. **218**(8): p. 2726-2742.
11. Gomez, J.M., et al., *Differential regulation of the proteome and phosphoproteome along the dorso-ventral axis of the early Drosophila embryo.* Elife, 2024. **13**.
