# Supplementary material for: Highly dynamic mechanical transitions in embryonic cell populations during Drosophila gastrulation
Source: Nat Commun. 2025 Jul 14;16:6473. doi: 10.1038/s41467-025-61702-4 (PMC12259986; doi:10.1038/s41467-025-61702-4)
Supplement: Supplementary file 2 — Description of Additional Supplementary Files [file 41467_2025_61702_MOESM2_ESM.pdf]

Description of Additional Supplementary Files

Supplementary Video 1
Cross-sectional live imaging of a *Drosophila* embryo from the onset of gastrulation (stage 5b) until the initiation of mesodermal EMT (stage 8). Key morphogenetic events that occur in cells distributed along the DV axis are indicated in the upper right corner. Membranes were labelled with a CaaX-eGFP (grayscale) transgene. The depth of the furrow increases with the progression of VFF. Imaging was conducted in a 2-photon microscope, and embryos were mounted in agar, vertically with respect to the glass bottom of the petri dish. The imaging was conducted approximately 180 µm from the posterior side of the embryo, and three consecutive planes were max-projected. Dorsal is top, ventral is bottom. Scale bars are 50 µm.

Supplementary Video 2
Live imaging of the dorsal side of a *Drosophila* embryo describing the squamous morphogenesis of the dorsal ectoderm and the amnioserosa cell populations using our custom-built Selective Plane Illumination Microscopy (SPIM) microscope coupled to the LSBM. Membranes were labelled with CaaX-eGFP (magenta) and nuclei were labelled with H2Av-mRFP (green) transgenes. Top left and bottom panels show a superficial (max-projection of 4 slices), in which the process of cellular stretching is appreciated. Top right panel shows the YZ resliced volume (1 slice), in which the changes in cellular morphology along the apical-basal axis can be seen. In top left and bottom panels anterior is left and ventral is top-bottom. In the top-right panel, dorsal is top. Scale bars are 50 µm.

Supplementary Video 3
Brillouin shift (BS, bottom) maps of the ventral side of the embryo (ventral is top), showing the dynamic changes in mechanical properties of the mesoderm from the onset of VFF (stage 5b) through mesodermal EMT (early stage 8). Imaging was performed on living embryos carrying transgenes that label membranes (CaaX-eGFP, top) and nuclei (H2Av-mRFP, centre). Video was assembled by YZ re-slicing the original raw volume and averaging two consecutive slices. Region shaded in yellow in CAAX-eGFP corresponds to the mesoderm cells that were used for quantification. Scale bar is 50 µm.

Supplementary Video 4
Colocalisation between the transient, high Brillouin shift (BS, bottom) measured in the mesoderm during VFF and lipid droplets (top). Imaging was performed on a YFP protein-trap transgenic line for the gene *lsd-2*, whose product binds to lipid droplets. Video was assembled by YZ re-slicing the original volume and averaging two consecutive slices. The ventral side of the embryo is top. Note that lsd2-YFP puncta do not colocalise with the high Brillouin shift in central mesoderm. Scale bar is 50 µm.

Supplementary Video 5
Brillouin shift (BS, bottom) maps of the lateral side of the embryo (lateral is top, ventral is left), showing the dynamic changes in mechanical properties of the neuroectoderm from the onset of VFF (stage 5b) until the neuroectoderm has fully displaced in the ventral (left) direction. Imaging was performed on living embryos carrying transgenes that label the membranes (CaaX-eGFP, top) and the nuclei (H2Av-mRFP, centre). Video was assembled by YZ re-slicing the original volume and averaging two consecutive slices. Region shaded in yellow in CAAX-eGFP corresponds to the neuroectoderm cells that were used for quantification. Scale bar is 50 µm.

Supplementary Video 6
Brillouin shift (BS, bottom) maps of the dorsal side of the embryo (dorsal is top), showing the dynamic changes in mechanical properties of the dorsal ectoderm and amnioserosa cells from the end of cellularisation (stage 5b) through the squamous morphogenesis of the dorsal ectoderm and amnioserosa (stage 7 and early stage 8). Imaging was performed on living embryos carrying transgenes that label the membranes (CaaX-eGFP, top) and the nuclei (H2Av-mRFP, centre). Video was assembled by YZ re-slicing the original volume and averaging two consecutive slices. Region shaded in yellow in CAAX-eGFP corresponds to the ectoderm cells that were used for quantification. Scale bar is 50 µm.

Supplementary Video 7
Brillouin shift (BS, bottom-left and bottom-right) maps of the ventral side of the embryo (ventral is top), showing the spatial distribution of pixels with the largest 4% Brillouin shift (thresholded signal in top-right panel; pixels enclosed by white line in bottom right panel) during VFF. Imaging was performed on living embryos carrying transgenes that label the membranes (CaaX-eGFP, magenta, top-left) and the nuclei (H2Av-mRFP, cyan, top-right). Video was assembled by YZ re-slicing the original raw volume and averaging two consecutive slices. Region shaded in yellow in CAAX-eGFP corresponds to the ectoderm cells that were used for quantification. Dotted white line indicates the basal boundary of the growing sub-apical compartment. Scale bar is 50 µm.

Supplementary Video 8
Colocalisation between the transient, high Brillouin shift (BS, bottom) measured in the central mesodermal cells during VFF, F-actin (labelled with a UtrophinABD-GFP transgene; grayscale; top) and non-muscle myosin light chain (labelled with a sqh-mCherry transgene; grayscale ; centre). Note the transient high Brillouin shift distributed across a larger tissue section than apical actomyosin. Video was assembled by YZ re-slicing the original volume and averaging two consecutive slices. Scale bar is 50 µm.

Supplementary Video 9
Mid-sagittal Brillouin shift (BS, bottom) maps of the dorsal side of the embryo (dorsal is top, anterior is left), showing the dynamic changes in mechanical properties during dorsal fold formation (DFF) from the end of cellularisation (stage 5b) until the end of posterior midgut (PMG) invagination (stage 8). Imaging was performed on living embryos that carry a transgene that labels the membranes (Gap43-mCherry; grayscale; top). Video was assembled by YZ re-slicing the original volume and averaging two consecutive slices. Anterior and posterior folds position along the anterior-posterior axis are indicated by arrowheads during the progression of DFF. Scale bar is 50 µm.

Supplementary Video 10
Brillouin shift (BS, bottom) maps of the ventral side of the embryo (ventral is top), showing the effect of Colcemid treatment (concentration: 1mg/ml in PBS; right panels) on the dynamic increase in BS measured within central mesodermal cells during VFF (PBS control, left panels; cells shaded in yellow). Imaging was performed on living embryos that carry a transgene that labels the membranes (Gap43-mCherry; grayscale; top). To deliver Colcemid, embryos were permeabilised (see Methods). Video was assembled by YZ re-slicing the original volume and averaging two consecutive slices. Region shaded in yellow in CAAX-eGFP corresponds to the mesoderm cells that were used for quantification. Scale bar is 50 µm.

Supplementary Video 11
Live imaging of EB1-GFP (grayscale) in embryos undergoing VFF, using a spinning disk microscope. Ventral is top. The sub-apical compartment in central mesoderm is enclosed by yellow-dashed shapes, at the onset of VFF and during fold formation. Video was assembled by

YZ re-slicing of the original volume and max-projecting 20 consecutive slices. Scale bar is 25 µm.

Supplementary Video 12
Brillouin shift (BS, bottom) maps of the ventral side of the embryo (ventral is top), showing the effect of *twist* loss of function (*twi*^{1} homozygous mutation; right panels) on the dynamic increase in BS measured within central mesodermal cells during VFF (*twi*^{1} heterozygous mutation; left panels). Imaging was performed on living embryos that carry a transgene that labels the membranes (CaaX-mScarlet; grayscale; top). Video was assembled by YZ re-slicing the original volume and averaging 2 consecutive slices. Scale bar is 25 µm. Scale bar is 50 µm.

Supplementary Video 13
Video of a simulation where the sub-apical regions of both central and peripheral cells are soft (λ_{sA}=0) leading to failure of ventral furrow formation. Sub-basal stiffness of all mesodermal cells is set to λ_{sB}=50. Video corresponds to the first panel of Fig. 5e'. Simulated time from 0 to 8.6min.

Supplementary Video 14
Video of a simulation where the sub-apical regions of central cells are soft ($\lambda_{sA,c}$=0) leading to excess elongation along their apical-basal axis and failure of ventral furrow formation. Sub-apical stiffness of peripheral cells is set to $\lambda_{sA,p}$=90, and sub-basal stiffness of all mesodermal cells is set to λ_{sB}=50. Video corresponds to the second panel of Fig. 5e'. Simulated time from 0 to 8.6 min.

Supplementary Video 15
Video of a simulation where the sub-apical region of central cells is stiff ($\lambda_{sA,c}$=110), but the peripheral cells are soft ($\lambda_{sA,p}$=0). The high deformability of the peripheral cells prevents the furrow from ingressing further (compare with Supplementary Video 16). Sub-basal stiffness of mesodermal cells is set to λ_{sB}=50. Video corresponds to the third panel of Fig. 5e'. Simulated time from 0 to 8.6min.

Supplementary Video 16

Video of a simulation where the longitudinal stiffness of the sub-apical regions of both central and peripheral cells is high ($\lambda_{sA,c}$=110 and $\lambda_{sA,p}$=90) leading to a deeper ventral furrow. Sub-basal stiffness of mesodermal cells is set to λ_{sB}=50. Video corresponds to the last panel of Fig. 5e'. Simulated time from 0 to 8.6 min.
