# Supplementary material for: Highly dynamic mechanical transitions in embryonic cell populations during Drosophila gastrulation
Source: Nat Commun. 2025 Jul 14;16:6473. doi: 10.1038/s41467-025-61702-4 (PMC12259986; doi:10.1038/s41467-025-61702-4)
Supplement: Supplementary file 19 — Reporting Summary [file 41467_2025_61702_MOESM19_ESM.pdf]

nature portfolio

Corresponding author(s): Gómez, Juan Manuel ; Prevedel, Robert

Last updated by author(s): May 21, 2025

Reporting Summary

Nature Portfolio wishes to improve the reproducibility of the work that we publish. This form provides structure for consistency and transparency in reporting. For further information on Nature Portfolio policies, see our Editorial Policies and the Editorial Policy Checklist.

Statistics

For all statistical analyses, confirm that the following items are present in the figure legend, table legend, main text, or Methods section.

n/a	Confirmed	
☐	☒	The exact sample size (*n*) for each experimental group/condition, given as a discrete number and unit of measurement
☐	☒	A statement on whether measurements were taken from distinct samples or whether the same sample was measured repeatedly
☐	☒	The statistical test(s) used AND whether they are one- or two-sided<br>*Only common tests should be described solely by name; describe more complex techniques in the Methods section.*
☒	☐	A description of all covariates tested
☐	☒	A description of any assumptions or corrections, such as tests of normality and adjustment for multiple comparisons
☐	☒	A full description of the statistical parameters including central tendency (e.g. means) or other basic estimates (e.g. regression coefficient) AND variation (e.g. standard deviation) or associated estimates of uncertainty (e.g. confidence intervals)
☐	☒	For null hypothesis testing, the test statistic (e.g. *F*, *t*, *r*) with confidence intervals, effect sizes, degrees of freedom and *P* value noted<br>*Give P values as exact values whenever suitable.*
☒	☐	For Bayesian analysis, information on the choice of priors and Markov chain Monte Carlo settings
☒	☐	For hierarchical and complex designs, identification of the appropriate level for tests and full reporting of outcomes
☒	☐	Estimates of effect sizes (e.g. Cohen's *d*, Pearson's *r*), indicating how they were calculated

Our web collection on statistics for biologists contains articles on many of the points above.

Software and code

Policy information about availability of computer code

Data collection	For statistical analyses we used: Prism GraphPad 8-10. For image analyses: Fiji/ImageJ. To run the code for MT alignment: Matlab 2019b.
Data analysis	We used the publicly available and open-source CompuCell3D software to run the computer simulations. The code for the model can be found at the public repository https://github.com/abhisha-ramesh/Gastrulation_Model.

For manuscripts utilizing custom algorithms or software that are central to the research but not yet described in published literature, software must be made available to editors and reviewers. We strongly encourage code deposition in a community repository (e.g. GitHub). See the Nature Portfolio guidelines for submitting code & software for further information.

Data

Policy information about availability of data

All manuscripts must include a data availability statement. This statement should provide the following information, where applicable:

- Accession codes, unique identifiers, or web links for publicly available datasets
- A description of any restrictions on data availability
- For clinical datasets or third party data, please ensure that the statement adheres to our policy

Methods section, Physical Model of Gastrulation subsection, it reads: ' The code for the simulations is publicly available at https://github.com/abhisha-ramesh/Gastrulation_Model.'

Research involving human participants, their data, or biological material

Policy information about studies with human participants or human data. See also policy information about sex, gender (identity/presentation), and sexual orientation and race, ethnicity and racism.

Reporting on sex and gender	NA
Reporting on race, ethnicity, or other socially relevant groupings	NA
Population characteristics	NA
Recruitment	NA
Ethics oversight	NA

Note that full information on the approval of the study protocol must also be provided in the manuscript.

Field-specific reporting

Please select the one below that is the best fit for your research. If you are not sure, read the appropriate sections before making your selection.

☒ Life sciences ☐ Behavioural & social sciences ☐ Ecological, evolutionary & environmental sciences

For a reference copy of the document with all sections, see nature.com/documents/nr-reporting-summary-flat.pdf

Life sciences study design

All studies must disclose on these points even when the disclosure is negative.

Sample size	Sample size was determined based on resources availability and the observed low variability across all quantifications. Therefore, we determined that was sufficient an N = 5 experimental units for all experiments with the sole exception of experiments used to assemble Figure 4 panels c-e, in which the N = 3. However, because we are aware the N of independent experiments is small, we showed for every quantification the values (or curve) for each embryo separately, and additionally, the averaged values (or curves).
Data exclusions	The curves shown in Fig. 2f were assembled using 7 out of the 9 cells quantified throughout squamous morphogenesis. In this panel, we plot an average time course of the cell-by-cell quantification of the Brillouin shift and the length of the apical-basal and stretching axes. Most of the cells displayed similar dynamics (7/9), however two cells reached the largest circularity value (when cells became largely isotropic) at different times to the rest. Therefore, we established an inclusion/exclusion criteria to assemble an average time course: we included those cells that acquired the largest circularity value between 00:42:20 and 01:07:44 hours, corresponding to the 10th and 90th percentile of the distribution of maximum circularity times. In these 7/9 cells, the maximum circularity value was measured at a median of 00:53:22 min, and this value was used in Fig. 2f to set the boundary between phase 1 and phase 2. This exclusion/inclusion criteria for the curves shown in Fig 2 panel f is detailed in the Methods section, 'Mask generation and measurements within region of interest' subsection of the manuscript.
Replication	Replication was successful with a measurable (low) degree of variability, which was reflected using either standard error of the mean or the 1st and 3rd quartiles of the distribution of points.
Randomization	NA
Blinding	NA

Reporting for specific materials, systems and methods

We require information from authors about some types of materials, experimental systems and methods used in many studies. Here, indicate whether each material, system or method listed is relevant to your study. If you are not sure if a list item applies to your research, read the appropriate section before selecting a response.

Materials & experimental systems

n/a	Involved in the study
x	Antibodies
x	Eukaryotic cell lines
x	Palaeontology and archaeology
	x Animals and other organisms
x	Clinical data
x	Dual use research of concern
x	Plants

Methods

n/a	Involved in the study
x	ChIP-seq
x	Flow cytometry
x	MRI-based neuroimaging

Animals and other research organisms

Policy information about studies involving animals; ARRIVE guidelines recommended for reporting animal research, and Sex and Gender in Research

Laboratory animals	Drosophila melanogaster embryos from reported transgenic and/or mutant lines.
Wild animals	This study made no use of wild animals.
Reporting on sex	NA
Field-collected samples	This study made no use of field-collected samples.
Ethics oversight	No need of ethics oversight for Drosophila melanogaster embryos.

Note that full information on the approval of the study protocol must also be provided in the manuscript.

Plants

Seed stocks	*Report on the source of all seed stocks or other plant material used. If applicable, state the seed stock centre and catalogue number. If plant specimens were collected from the field, describe the collection location, date and sampling procedures.*
Novel plant genotypes	*Describe the methods by which all novel plant genotypes were produced. This includes those generated by transgenic approaches, gene editing, chemical/radiation-based mutagenesis and hybridization. For transgenic lines, describe the transformation method, the number of independent lines analyzed and the generation upon which experiments were performed. For gene-edited lines, describe the editor used, the endogenous sequence targeted for editing, the targeting guide RNA sequence (if applicable) and how the editor was applied.*
Authentication	*Describe any authentication procedures for each seed stock used or novel genotype generated. Describe any experiments used to assess the effect of a mutation and, where applicable, how potential secondary effects (e.g. second site T-DNA insertions, mosiacism, off-target gene editing) were examined.*
